# On the Sanitary and Social Conditions of the English Poor

**Published:** 1855

**Authors:** 


### ORIGINAL COMMUNICATIONS.

---

ON THE

### SANITARY AND SOCIAL CONDITIONS OF THE ENGLISH POOR.

By a PRACTISING PHYSICIAN.

No. I.

FROM the day on which I commenced my career as a practitioner to the present time, I have been in the constant habit of visiting and prescribing for the sick poor; so much so, that, on looking back, I can almost remember those days (I might call them holidays) in which no such labours were performed. At first I attended on this large class of the community from necessity; I do so now, in some degree, from habit, and in a greater degree from choice. During the period of my connection with the poor, many opportunities have of course occurred to me for observing their social and sanitary condition; and I have some hopes of unfolding in these pages a few facts which, if already known, cannot be too often confirmed, until such time as their removal renders their further confirmation impossible, and the recollection of them unnecessary.

I have tried for some time past to reduce the thoughts I have on this subject to some kind of order, and to express them systematically, as the technical and hard-fact men express it. But this end I find impossible to attain; my mind obviously is not made for it, and the rule and the plummet paralyse me at first sight. I shall therefore travel on in my own way, with the mere intimation that whatever is badly expressed is well meant.

Some one will perhaps ask me, whom I mean by the poor? If such an one wishes me to answer the question on a kind of mathematical principle, I confess myself at once standing before a verbal *pons asinorum*, which it is beyond my ability to cross. Speaking generally, however, I may say that my chief reference will be to the lowest section of the masses; to men and women who, whatever their occupation may be, have to gain their bread by the hand rather than the head; who inhabit the most wretched parts of our cities and towns; from whom the great stream of education is cut off, or supplied in a vitiated form; and who, from the first to the last day of the year, are, as a general rule, employed for two-thirds of each twenty-four hours in securing the necessaries of existence alone, regardless almost entirely of the necessaries of health and happiness.

Glancing over the wide field before me, I scarcely know where to break ground. I think, however, as I have no system to enthral me, I will follow the example of voyagers, and, before describing the people of whom I write, take a telescopic view of the places they inhabit.

It has often occurred to me that, if the whole of this island could be obliterated for some thousand years or so, like Pompeii or Herculaneum, some antiquarian society of a future day might, if fortunate enough to turn up this remnant of the old world, spend many years in discussing what kind and manner of animals could ever have lived in some of the erections which we call human dwellings. Buckingham Palace or Princes Terrace might, it is true, be comprehended as having been the habitations of mankind; but what would be made of the wynds of Glasgow, the cellars of Liverpool, or the common lodging-houses in Somers-town, it is utterly impossible to say. The discussion would only end, as far as I can see, by an unanimous decision that the buildings in question were the residences of some extinct race of animals of very small size, possessed of eyes peculiarly sensitive to light, of brains divested of olfactory nerves, and of tastes but one degree higher than those which prevail in those classes of creation where mere instinct hold the place of mind. I do not think I exaggerate in making this speculation. I took an odd fancy some time ago to compare certain buildings or dwelling-houses of a few inferior animals with certain similar buildings or dwelling-houses of the human family; and I can safely say that the experiment justifies the above idea. Let me give one example. A beaver, in building his tenement, instinctively takes special pains to make it healthy; he dams off the water from his dwelling by a convex wall; he makes a cabin some seven or eight feet in diameter; he divides this cabin into two compartments, the upper one of which serves as a living room, the lower as a storehouse; he leaves an opening for escape, which is blocked up by no barrier; and he allows plenty of chinks for ventilation in every part. When his residence is built, he encumbers it with no excrementitious matter; and there seems to exist a kind of beaver enactment, by which a certain number only of individuals shall live in one hut. For certain it is that, according to the number of inhabitants in any beaver community, a certain number of houses are built; and that lodging-house keeping and overcrowding are matters utterly unknown amongst this class of the living creation.

Let me now compare this beaver-dwelling with one or two human habitations which I have seen. I went to the top of a house in a densely populated part of London a few days ago, to see a poor woman suffering from inflammation of the lungs. Her single room was as much her distinct habitation as the beaver's is his, and was encumbered with very much more furniture; for there was a bedstead in it, which took up nearly half of its absolute size, besides two chairs, a table, and various cooking utensils suspended about the wall. I measured this room: it was nine feet square and six feet high, and, when divested of furniture, would probably contain four hundred and eighty-six cubic feet of air; but with the furniture and two persons in it, it would hold very little more than half this proportion of the life-sustaining element. Ventilation there was none: on the contrary, every crevice was carefully closed to make the place warm; and as to light, that too was reduced to a glimmer, for the window had a northern aspect, was overshadowed by buildings, was in size not more than two feet square, and was so thoroughly patched over with paper, that it looked like one of those windows which we were accustomed to see a short time since, in a state of barricade against the encroachments of the tax-collector.

I remember too, when taking charge of an union district, in an agricultural neighbourhood, being one day hailed as I rode past a miserable little hamlet, to see a very aged woman, whom I may here call Betty Fletcher, though that was not exactly her name. As I dismounted from my horse, a middle-aged person, a neighbour of the patient, took me quietly on one side, and begged me not to be taken by surprise, or to be alarmed at the appearance of the old woman, as she was very peculiar, and was a well known witch. Smiling at the remark, I followed my guide along a very dirty path, into a little hut, built at the foot of a muddy and foul pond. As I entered the hovel through a door about five feet high, I was certainly not a little astonished at the place itself and its remarkable inmate. The place was a thorough mud dungeon, and was, I should say, about three yards long, two yards wide, and five feet high in the centre, for the roof was so sloping that the eaves came nearly to the ground. There was no window, and the fireplace was, of all I have ever seen, the rudest. A small recess at the end of this place, forming so exact a miniature that it looked like a new-born progeny of the larger apartment, constituted the bed-room of my patient, who certainly did bear such a resemblance to a witch of olden time, that I doubt not her appearance alone would have committed her to the stake in the days of the witch-killing mania. She was in the hour and article of death; and I shall never forget, while I live, the sensations which this sight caused in me. I learned that she had lived in this hole for the long period of twenty years, neglected almost entirely by her neighbours, and visited often for weeks together by none save the relieving officer, who hated her, and who never brought her her weekly two shillings and ninepence and loaf of bread, without tacitly wishing that she might be robbed of the former, and choked by the latter.

I could bring forward many more instances of this kind, were it necessary: but I think these are sufficient to prove my beaver theory; and I am perfectly sure that no antiquarian of a thousand years hence would ever classify Betty Fletcher's hovel amongst the probable residences of the genus *homo.*

I need not enter into many illustrations on the subject of over-crowding in the small tenements of the poor. Let the lodging-house keeping records, as they sometimes ooze out at our police-courts, tell of this. I will give but one or two instances, and these only because they are amongst the most remarkable I have seen out of several hundreds.

The first was in a country village, overwhelmed with members of the labouring class. In this village there was a small closed-in court or terrace of houses, which was called by the natives "Little Britain," in order, as I suppose (for I never could get at any other reason), to illustrate, in two words, how large a number of people lived on a small isolated patch of ground. This court consisted of thirteen cottages, all in a row. There was a blank wall or fence of about their own height before them, and between the houses and the wall was a small "green," as it was called, which consisted in fact of thirteen fantastically shaped middens, composed of the most undefinable materials, linked together in a lank, irregular chain, emitting a most concentrated midden odour, and presenting no green material at all, except cabbage leaves, which, being themselves subject to change of colour in their dead state, did not add much to the prospect. This "green," moreover, was ornamented with several pigsties, each of which contained two or three inmates; and there were two other buildings connected with the houses, which it is unnecessary to describe by name, though they were most objectionable edifices in such a place, and constructed on a most unhealthy design. The houses were all joined together, and consisted of two floors,—the ground floor, which formed one room, and measured about nine feet square, and a bed-room floor of the same size, which was divided into two apartments. It will, perhaps, scarcely be credited that at the time of which I write, there were no fewer, in the shape of men, women, or children, than thirteen individuals in each of these houses. I mean rather, that the number of people living in this manner averaged thirteen for each house. How they were all packed at night, I could never define, and do not pretend to say. I used, however, to think that the labours of Morpheus must be very onerous in that particular spot; and if half the people had ever been found dead in their beds after some sultry summer's night, I do not see how a coroner's jury could have returned any other verdict than death by general suffocation.

Of course disease was peculiarly rife in this "Little Britain." The place was in fact a kind of ready-made, ready-furnished hospital; and if any medical student should wish to see practice in a hospital of his own, he could not do better than take a set of houses of a similar kind, and overstock them in a similar way. He would then soon have work enough for his "prentice han'," I confidently assure him.

The diseases peculiar to these localities, in the agricultural districts, are most common amongst the women and young children,—amongst the latter especially. This is explainable on very simple principles. The men are out the greater part of the day at their work, and the older children are either at work, at school, or at play. In this way they escape full half the penalty of a confined and vitiated atmosphere; while the infants and the women, who remain at home, are continually exposed to the said penalty, and are always ailing and miserable.

This example of over-crowding in a rural village is flagrant enough, but is as nothing compared with what occurs in some large towns and cities. If I wished, I could fill page after page with statistics on this point, all tending to show that men would be better living like cattle in dry meadows, without shelter or home, than in many of the habitations of our most thriving and important towns. One instance occurs to me, which it would be well to name. It may seem an exaggerated picture to people who live in the midst of plenty, and who have no idea whatever about the other half of the world; but it is no exaggeration,—it is but a type of many other similar instances with which the medical man soon becomes familiar.

The scene of this picture of misery is laid in a large northern city. There are in this city three long narrow courts or streets, which take a waved direction, and lie almost parallel to each other, like three huge venomous serpents with a poison-bag on every square inch of their bodies. These courts are of great length, and so narrow that a broomstick is easily stretched across from the window of one house to the corresponding window of the house opposite, a convenience which the genius of the place has turned to considerable advantage; since those who consider clean linen a luxury are enabled, by the simple mechanical appliance already named, to turn the court into a drying-ground, during their laundry performances. The houses of these courts are usually high; the original proprietor having discovered that, as the vault of heaven was every man's property, he might increase his tenantable possessions to a great extent, and at little cost, in that direction. At one time, perhaps, the streets of the courts might have been paved; but the feet of the multitude have so long since obliterated any kind of pavement, that it is quite impossible to say whether or not such a convenience ever existed. To render the houses more richly fertile with human sorrows, the rooms are arranged on the bee-cell principle; *i. e.*, the architect has made it his especial study to make every room complete in itself, without any unnecessary expenditure of stone, mortar, or space. Thus contrived, each compartment contains every inconvenience for a distinct family, and becomes accordingly a special residence.

From all that I could learn, it would appear that the serpentine courts now being described have never during the whole period of their existence, received a direct visit from a sunbeam; and if such a beam were, by any accident, to throw itself into these chinks of streets, the people, I take it, would look upon the fact as a great phenomenon—the counterpart of an eclipse. However, there is no danger of this, unless the earth should begin to roll from north to south; for the long diameter of the courts runs in nearly the same direction as the zodiac, and the great luminary never gets high enough toward the zenith in these parts to stare straight down upon the earth beneath. It is a matter of importance to know also, that the winds can only freely ventilate these places from two opposite points, as there are no direct openings at a right angle with the main course.

As a matter of necessity these courts, serpents, or in more polite language, narrow streets, are very dens of disease—palaces of death; and it was in one of them that the instance of over-crowding I am about to relate occurred. At the time of the occurrence, an epidemic of measles was re-enacting King Herod amongst the infant population with considerable success. I was asked by a medical friend to see with him a patient whom he had attended in her accouchement, in one of the cells previously described. After passing up several flights of stairs, we came to the dungeon, where my friend in the performance of his duties had spent the previous night. Bear with me, gentle reader, as I raise the curtain. If you, unaccustomed to such sights, shudder, believe me that I, accustomed to them, shuddered then, and shudder now, as my pen proceeds. The den was small, low, dark, filthy; to the sight, disgusting; to the olfactory sense, sickening; to the ear, appalling. In the corner of the room furthest from the door, on dirty straw, lay the patient whom my friend had attended. By her side lay the luckless, wretched, new-born visitor of the earth. They were both but half living; and the little one, as if already wearied of its existence, was ever uttering a kind of mournful cry, which its half unconscious mother vainly tried to hush. In the opposite corner lay an old fat drunken woman, with her grand-daughter, both the victims of a loathsome skin disorder, and both complaining loudly, and in coarse terms, of their noisy neighbour, the unfortunate innocent. At the other end of the room, also on a bed of straw, with scanty coverings, lay five children, varying in age from three to eight years. They were huddled together, and were attended to by a wretched old woman, who, from habit, seemed to treat the whole with the most supreme indifference. These children were all suffering from the epidemic; and my friend informed me that during the night he had paid attention to their condition, and found that they had dangerous pulmonary symptoms. We stepped amongst them to see how they were. There were three very ill, two of whom were in a great measure unconscious; there was one dying—*there was one dead.* "Do you know," said I to the old woman looking on, "that this child is dead?" "Oh, yes, she knew it well enough; it died at seven o'clock, and she had sent word to the parochial authorities for a coffin, which would be there at five, and then the dead one might be removed from the living."

In the evening of the day upon which this scene occurred, I walked out of the city to a certain exquisite burial-place not far off, where the architect's and sculptor's arts stood out in full array; where flowers grew, and green boughs waved, and where every care was taken that the silent occupants of the place should not lie in too close proximity. As I viewed this care for the dead, the thought of the previous sight I had seen opened up a strange contrast; and I felt that, were the choice offered me to be forced either to half live in the den I had previously visited, or to lie in cold oblivion amongst the sleepers around me, I should prefer the last named alternative, with the deep quiet earth for my bed, and the waving boughs for my canopy.

*(To be continued.)*

